# Design of Electrostatic Nanocomplex of Semaglutide with Protamine and Zinc for Subcutaneous Prolonged Delivery

**DOI:** 10.3390/nano15181399

**Published:** 2025-09-11

**Authors:** In Gyu Yang, Jeong-Soo Kim, Myung Joo Kang

**Affiliations:** 1 College of Pharmacy, Dankook University, 119 Dandae-ro, Dongnam-gu, Cheonan 31116, Republic of Korea; dhakflzk@naver.com; 2 Dong-A ST Co., Ltd., Giheung-gu, Yongin 17073, Republic of Korea; js_kim@donga.co.kr

**Keywords:** semaglutide, electrostatic interaction, protamine sulfate, nanocomplex, lipophilicity, sustained release

## Abstract

The aim of this study was to design a poorly water-soluble electrostatic nanocomplex of semaglutide (SMG) with protamine sulfate (PS) and zinc ions (Zn) for prolonged subcutaneous delivery. Complexation of SMG with the cationic peptide PS increased the lipophilicity (logP) proportionally from −4.7 to 0.3, particularly in the presence of Zn. The optimized nanocomplex exhibited spherical morphology, an amorphous state, a particle size of 196.0 nm, and a zeta potential of −45.7 mV. In an in vitro dissolution test under sink conditions, native SMG showed rapid drug release with 98% dissolution within 24 h. In contrast, the nanocomplexes showed markedly delayed release, with a concentration-dependent relationship between PS/Zn contents and SMG release rate, exhibiting 19% drug release over 7 days in the optimized formula. These findings suggest that the proposed nanocomplex is a promising system for long-acting injectable delivery of SMG, potentially enhancing patient compliance in patients with obesity or type 2 diabetes.

## 1. Introduction

**Figure 1:**
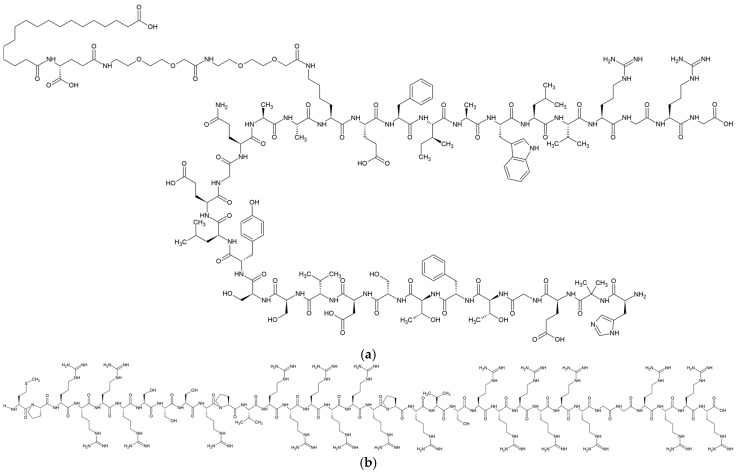
Chemical structure of SMG (**a**) and PS (**b**).

Semaglutide (SMG) (Figure 1a), a glucagon-like peptide-1 receptor agonist (GLP-1RA), has emerged as a pivotal therapeutic agent for type 2 diabetes and obesity due to its robust efficacy in glycemic control, weight reduction, and cardiovascular risk mitigation [1,2,3,4]. SMG is a 31-amino acid peptide with a molecular weight of 4113.58 Da, structurally derived from native GLP-1 by substitutions at positions 8 (alanine to α-aminoisobutyric acid) and 34 (lysine to arginine), and acylation of the lysine residue at position 26 with a C18 fatty diacid chain. These modifications enhance albumin binding, impede enzymatic degradation, and significantly extend the peptide’s half-life in circulation [5,6,7,8,9,10]. Clinically, SMG is available in once-weekly subcutaneous (SC) injection and once-daily oral tablet formulations [11]. The SC injection (e.g., Ozempic^®^) is a sterile, isotonic aqueous solution at pH 7.4, containing disodium phosphate, propylene glycol as a co-solvent, and phenol as a preservative [12]. Following SC injection, the SMG peptide at the injection site binds reversibly to endogenous albumin in the interstitial fluid and plasma, protecting the peptide from degradation by dipeptidyl peptidase-4 (DPP-4). Accordingly, the drug exhibits a dose-proportional pharmacokinetic profile in healthy subjects, with an elimination half-life of approximately 7 days, supporting its once-weekly dosing regimen [13]. Standard dosing initiates at 0.25 mg weekly, titrated up to 2 mg depending on glycemic response and tolerability [12]. To extend the dosing interval and enhance treatment adherence, different pharmaceutical approaches have been explored. These include encapsulation into polymeric microspheres using double-emulsion or microfluidic techniques, and tethering peptides to biodegradable matrices via cleavable linkers [5,9]. Although preclinical results demonstrated promising therapeutic efficacy, these strategies are challenging due to low loading efficiency, drug release control, drug instability, and difficulties in the scale-up process [14,15,16,17].

Protamine sulfate (PS) (Figure 1b), a cationic polypeptide derived from salmon sperm, is widely used in drug delivery due to its ability to form stable electrostatic complexes with anionic therapeutic agents [18,19,20,21]. In insulin formulations, PS binds to insulin hexamers via hydrogen bonding, hydrophobic interactions, and salt bridges involving arginine residues. The electrostatic complexation stabilizes the insulin hexamer and slows its dissociation into monomers, thereby extending the duration of insulin action after SC injection [18]. Molecular dynamics studies revealed that these complexes remain dynamically stable over time, maintaining the hexametric state and enabling sustained monomer release over 12–24 h [19]. Moreover, protamine–oligonucleotide nanoparticles loaded with secretoneurin were engineered using a stepwise titration method, enabling delayed biodistribution kinetics and exhibiting extended retention at intramuscular injection sites in vivo [20,21].

Drawing on these insights, we designed a novel electrostatic nanocomplex of SMG with cationic PS and Zn for SC sustained delivery. We assumed that the solid-state, poorly water-soluble SMG-PS nanocomplexes suspended in the aqueous vehicle slowly dissolved at the injection site following SC administration. Subsequently, SMG molecules dissolved at the injection site bind to endogenous albumin and resist DPP-4 degradation, providing a further extended pharmacokinetic profile. To design the novel system, the ratios of SMG, PS, Zn, and suspending agents were tuned to adjust lipophilicity, aqueous solubility, particle size, zeta potential, and dispersibility. The prepared nanocomplexes were further characterized by morphology, crystallinity, and the interaction between each component using Fourier-transform infrared (FT-IR) spectroscopy. Moreover, in vitro release profiles of nanocomplexes depending on SMG, PS and Zn ratios were evaluated under sink conditions using a dialysis-bag method.

## 2. Materials and Methods

### 2.1. Materials

SMG drug powder was kindly provided by Dong-A Pharm Co., Ltd. (Seoul, Republic of Korea). PS was purchased from Fujifilm Wako Pure Chemical Corporation (Osaka, Japan). Zinc chloride, sodium carboxymethyl cellulose (Na.CMC, molecular weight: 90,000 g/mol), propylene glycol, sodium phosphate dibasic dihydrate, trifluoroacetic acid, and *n*-octanol were obtained from Sigma-Aldrich (St. Louis, MO, USA). Polyoxyl castor oil (Kolliphor^®^ ELP) was obtained from BASF (Ludwigshafen, Germany). The dialysis membrane of 1000 kDa (MWCO) was purchased from Spectra/Por^®^, Spectrum Laboratories Inc. (Rancho Dominguez, CA, USA), Acetonitrile (HPLC grade) was supplied by Samchun Pure Chemical Co., Ltd. (Pyeongtaek-si, Republic of Korea), and all other reagents used were of analytical grade and used without further purification.

### 2.2. Preparation of SMG-PS-Zn Complexes

SMG–PS–Zn complexes were prepared by mixing aqueous solutions of oppositely charged peptides under controlled conditions [22]. Initially, 0.2 g of propylene glycol was dissolved in 10 mL of distilled water to serve as an isotonic agent. Na.CMC (0–10 mg/mL) and Kolliphor ELP (0–15 mg/mL) were then added to the propylene glycol solution as suspending agents. SMG (10.72 mg) was introduced into 2 mL of the resulting vehicle and vortexed for 30 min to obtain a clear solution. Separately, PS (0, 0.4, 0.8 and 1.2 mg/mL) and ZnCl_2_ (0 and 0.5 mg/mL) were dissolved in distilled water in a second vial. The PS/Zn^2+^ solution was added dropwise to the SMG solution and stirred at 200 rpm for 30 min to induce electrostatic complexation. The weight ratios of SMG, PS, Zn, Kolliphor ELP, and Na.CMC in the formulation were varied within the range of 5:36:0.4–1.2:0–0.5:0–10:0–15 (*w*/*w*). During the process, pH was adjusted to 7.4 using 0.1 N HCl or NaOH. The final suspensions were stored at 4 °C for subsequent experiments.

### 2.3. Determination of Lipophilicity of SMG-PS-Zn Complexes

The prepared SMG–PS–Zn complex suspensions were stored at −80 °C for 24 h, then freeze-dried using a lyophilizer (TFD 8501, Ilshinbiobase Co. Ltd., Dongducheon-si, Republic of Korea) at −80 °C and under a vacuum of <10 mTorr for 24 h to obtain dried samples. Then, the octanol–water partition coefficient (logP) was determined using the classical shake-flask method [23]. Equal volumes of n-octanol and distilled water were mutually saturated by vigorous mixing and phase separation. Ten milligrams of freeze-dried complexes were placed in a 20 mL scintillation vial, followed by the addition of 10 mL each of the pre-saturated octanol and water phases. The vial was sealed and stirred at 400 rpm for 24 h to reach equilibrium. After incubation, the mixture was centrifuged at 13,000 rpm to separate the phases. The octanol phase was diluted 10-fold with methanol, and the aqueous phase was diluted 10-fold with the mobile phase. Drug concentrations in both phases were quantified using high-performance liquid chromatography (HPLC) analysis, and logP was calculated as the logarithm of the ratio of drug concentration in octanol to that in water.

### 2.4. HPLC Analysis

The concentration of SMG in the complex suspension was quantified by reversed phase-HPLC, with minor modifications to previously reported methods [24,25]. Shimadzu HPLC system consisted of a pump (Model 515), UV–Vis detector (Model 486), and autosampler (Model 717 Plus), and fitted with a Capcellpak C18 column (4.6 × 250 mm, 5 μm, 30 °C). Prior to analysis, samples were diluted 20-fold with the mobile phase, filtered through a 0.22 μm PVDF membrane, and injected into the HPLC system. The mobile phase consisted of acetonitrile, distilled water, and trifluoroacetic acid (55:45:0.1, *v*/*v*/*v*), delivered at a flow rate of 1.0 mL/min. The injection volume was 20 μL, and detection was performed at 230 nm. SMG was eluted at approximately 7.8 min under these conditions. The calibration curve was linear across the range of 1–1000 μg/mL (y = 6682.3x + 17,945, r^2^ = 0.999). The limits of detection and quantification were 0.523 μg/mL and 1.569 μg/mL, respectively.

### 2.5. Particle Size and Zeta Potential of SMG-PS-Zn Complexes

The particle size and polydispersity index (PDI) of SMG-loaded nanocomplexes were determined using a Zetasizer Nano (Malvern Instruments Ltd., Malvern, UK) equipped with a 4 mW He–Ne laser (633 nm) operating at a scattering angle of 90°. Samples were diluted 10-fold with distilled water and placed in disposable cuvettes for measurement. Zeta potential was assessed using the same instrument at 25 °C. For zeta potential analysis, samples were diluted 10-fold with distilled water and loaded into specialized capillary cells. The dispersion medium was set to distilled water, with parameters adjusted as follows: viscosity of 0.8872 cP, dielectric constant of 78.5, and refractive index of 1.330. Each measurement comprised 15 runs and was performed in triplicate (n = 3).

### 2.6. Dispersibility of the Complexes After Centrifugation

The physical stability of the SMG-loaded complexes was evaluated by assessing their dispersibility after centrifugation [26]. Briefly, 1 mL of each sample was transferred into an Eppendorf (EP) tube and centrifuged at 13,000 rpm for 10 min at 4 °C. Following centrifugation, the sediment was gently redispersed by manual shaking for 10 min. The redispersed samples were pretreated identically to the original formulations and analyzed via HPLC. Redispersed (%) was calculated as follows: Redispersed (%) = C_redispersed_/C_intial_ × 100, where C_redipersed_ is the drug concentration in the redispersed sample and C_initial_ is the drug concentration prior to centrifugation.

### 2.7. Solid-State SMG (%) After Addition into Phosphate-Buffered Saline (PBS)

To simulate the remaining SMG in solid-state form still at the injection site following SC injection, SMG–PS–Zn complex formulations were added to PBS as a simulated environment, and the proportion of SMG in solid-state (%) was quantified [27]. Specifically, 1 mL of each complex sample was added dropwise to 5 mL of PBS, followed by mixing with a multivortexer at 2000 rpm for 2 h. Subsequently, 1 mL of each PBS sample was centrifuged at 13,000 rpm for 10 min at 4 °C. The supernatant was collected, pretreated, and analyzed by HPLC. The percentage of SMG remaining in solid-state form was calculated using the following equation: Solid-sate SMG (%) = (C_total_ − C_supernatant_)/C_total_ × 100, where C_total_ represents the total concentration of SMG in PBS, and C_supernatant_ is the SMG concentration in the supernatant after centrifugation.

### 2.8. FT-IR Spectroscopy

FT-IR spectroscopy was employed to investigate molecular interactions by analyzing characteristic peak shifts and changes in band intensities among individual components and their electrostatically assembled complexes [28]. The samples included SMG raw powder, PS raw powder, and different complexes prepared at defined weight ratios: SMG–PS (5.36:1.2, *w*/*w*), SMG–PS–Zn (5.36:1.2:0.5, *w*/*w*/*w*), and SMG–PS–Zn–CMC (5.36:1.2:0.5:2.5, *w*/*w*/*w*/*w*). Each complex sample was prepared and lyophilized as described in Section 2.3. FT-IR spectra were acquired using the potassium bromide (KBr) pellet method. Approximately 1–2 mg of the sample was finely ground with 200 mg of spectroscopic-grade KBr, and the mixture was compressed into a transparent pellet using a hydraulic press. Spectral data were then recorded in the range of 4000–400 cm^−1^ at a resolution of 4 cm^−1^, with particular attention given to functional group regions including hydroxyl, amine, and amide bands to identify potential hydrogen bonding, ionic interactions, and metal coordination.

### 2.9. Morphology and Physical Characteristics

#### 2.9.1. Morphological Observation

The morphological characteristics of the SMG-loaded complexes were evaluated using a scanning electron microscope (SEM, Helios 5, Thermo Fisher Scientific Inc., Waltham, MA, USA). Complexes prepared with varying ratios of SMG, PS, and Zn were centrifuged at 13,000 rpm for 10 min at 4 °C. After removing the supernatant, the pellets were carefully placed onto carbon adhesive tape and air-dried at room temperature for 24 h. The dried samples were then mounted onto aluminum stubs and sputter-coated with a thin platinum layer to enhance conductivity and prevent charging. SEM imaging was performed at an acceleration voltage of 5 kV to examine the surface morphology of the nanoparticles.

#### 2.9.2. X-Ray Diffraction (XRD) Analysis

The solid-state crystallinity of SMG powder and the SMG complexes with different ratios of SMG, PS, and Zn was analyzed using an X-ray diffractometer (Ultima IV, Rigaku Corporation, The Woodlands, TX, USA). Dried samples were prepared via the lyophilization method described in Section 2.3. Intact or lyophilized powders were then mounted onto flat aluminum sample holders, and diffraction patterns were recorded using CuKα radiation (λ = 1.54 Å) at an operating voltage of 40 kV and a current of 35 mA. Scans were performed over a 2θ range of 5° to 60° with a step size of 0.02° and a scanning rate of 2°/min.

#### 2.9.3. Differential Scanning Calorimeter (DSC) Analysis

Thermal analysis of SMG powder and SMG–PS–Zn complexes was conducted using a differential scanning calorimeter (DSC; Mettler Toledo International Inc., Greifensee, Switzerland) to assess physical state transitions after electrostatic complexation. Approximately 5 mg of SMG raw material and each lyophilized formulation were weighed and sealed in a standard aluminum pan. Samples were scanned over a temperature range of 0–300 °C at a heating rate of 10 °C/min under a nitrogen purge (flow rate: 50 mL/min) at ambient pressure.

### 2.10. In Vitro Dissolution Profile of SMG-Loaded Nanocomplexes

Drug release from nanocomplexes was evaluated using a dialysis bag diffusion method [29,30]. Each formulation containing SMG (5.36 mg/mL) was sealed in a dialysis bag (molecular weight cut-off: 1000 kDa) and immersed in 200 mL of phosphate-buffered saline (PBS, pH 7.4), which served as the release medium. The system was maintained at 37 ± 0.5 °C and gently agitated at 100 rpm using a shaking incubator (BF-60SIR, Biofree Co., Ltd., Seoul, Republic of Korea). At predetermined time points (0.5, 1, 2, 4, 8, 12, and 24 h, and then every 24 h for up to 7 days), 1 mL aliquots of the external medium were withdrawn for analysis. Each aliquot was centrifuged at 13,000 rpm for 10 min at 4 °C, and the resulting supernatant was diluted 2-fold with mobile phase prior to quantification by HPLC. After sampling, an equal volume of pre-warmed PBS was added to maintain sink conditions.

### 2.11. Statistical Analysis

All experiments were performed in triplicate or more. Data are presented as mean ± standard deviation (SD). Statistical analyses were conducted using an unpaired two-tailed Student’s *t*-test or one-way analysis of variance (ANOVA), followed by Tukey’s post hoc test. A *p*-value of less than 0.05 was considered statistically significant.

## 3. Results and Discussion

### 3.1. Effect of PS and Zn Concentration on logP and Solubility of SMG-PS-Zn Complexes

To construct a novel SC controlled-release system for SMG, we investigated the formation of an electrostatic complex comprising SMG, PS, and Zn. Cationic peptide–peptide complexes have recently attracted attention for their capacity to form stable, slowly dissolving depots via ionic interactions—an approach demonstrated in insulin and other peptide-based formulations [31,32,33]. We hypothesized that PS interacts electrostatically with the anionic residues of SMG, neutralizing its overall charge and promoting the formation of lipophilic electrostatic complexes. In addition, we hypothesized that Zn^2+^ ions further stabilize the electrostatic complex by coordinating with histidine or acidic residues of SMG. Previous protein structure analyses, including large-scale reviews of crystal structures, have shown that Zn^2+^ ions are predominantly coordinated by histidine and acidic residues (Asp/Glu) in peptides, typically adopting a tetrahedral geometry [34]. Furthermore, single-crystal X-ray diffraction studies of Zn-complexes containing bis-imidazole and carboxylate ligands have demonstrated that zinc forms direct bonds with both the nitrogen atoms of imidazole and the oxygen atoms of carboxylate groups [35]. This complexation strategy with PS and Zn is expected to provide insoluble SMG nanoparticles and aggregates at the injection site, enabling sustained SC release of the peptide. Following dissolution, SMG binds to endogenous albumin and evades degradation by DPP-4, thereby further prolonging its pharmacokinetic profile.

In this study, to minimize potential adverse effects, the amount of PS used in the SMG complex formulation corresponding to a two-week dose (2.68 mg of SMG) was minimized to 0.6 mg. PS administration is associated with potential risks, including severe hypotension, cardiovascular collapse, and pulmonary complications such as noncardiogenic edema and catastrophic vasoconstriction [36,37]. The maximum recommended single intravenous dose of PS is 50 mg over 10 min [37]. SC injection of PS has been also adopted in clinical practice, including in the use of Neutral Protamine Hagedorn (NPH) insulin [38]. Clinically, NPH insulin is typically administered once or twice daily at doses ranging from 0.5 to 1.0 units per kilogram of body weight [38]. It is formulated as a complex with protamine at a weight ratio of approximately 10:1 (insulin:protamine) [38], indicating that a 100-unit dose of insulin contains approximately 0.35 mg of PS per single dosing. Moreover, subcutaneously injected fragmin/protamine complexes (~0.6 mg PS per 0.20 mL) were tolerable in mice, with no injection-site bleeding or other adverse effects up to 10 days [39]. Therefore, the PS dose used in this study corresponds to approximately 1.2% of the dose associated with intravenous toxicological outcomes and is comparable to, or lower than, the established single SC dosing levels tested in preclinical or clinical studies.

**Figure 2:**
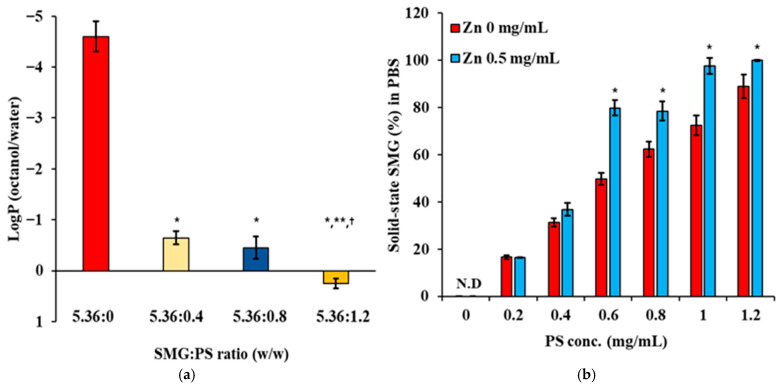
Changes in lipophilicity and solubility of SMG in aqueous media following complexation with PS in the absence and/or presence of Zn. (**a**) LogP (octanol/distilled water) values of SMG–PS complexes at varying SMG:PS ratios (5.36:0–5.36:1.2 mg/mL) in the presence of Zn (0.5 mg/mL), measured at 25 °C (**b**) Percentage of SMG remaining in solid-state form following the addition of complexes into phosphate-buffered saline (PBS), as a function of PS concentration (0–1.2 mg/mL) and Zn ion content (0 or 0.5 mg/mL) with 5.36 mg/mL of SMG, measured at 25 °C. Notes: Data represents mean ± SD (n = 3). N.D. means ‘not detected’. In panel (**a**), significant differences compared with 5.36:0 (* *p* < 0.05), 5.36:0.4 (** *p* < 0.05), and 5.36:0.8 (^†^ *p* < 0.05), respectively. In panel (b), significant difference compared with Zn 0 mg/mL (* *p* < 0.05).

Lipophilicity measurements revealed a dramatic shift in the physicochemical properties of SMG upon complexation with PS (Figure 2a). Native SMG exhibited high hydrophilicity, with a logP value of approximately −4.7, consistent with its peptide nature and abundance of polar residues. Complexation with PS at 0.4 mg/mL increased the logP to −0.7, representing a 4-log unit increase in lipophilicity. Further increases in PS concentration to 0.8 mg/mL and 1.2 mg/mL resulted in logP values of −0.2 and 0.3, respectively, indicating a concentration-dependent enhancement in lipophilicity. This substantial increase confirms successful electrostatic interaction between SMG and PS.

The increase in lipophilicity markedly reduced the aqueous solubility of the SMG–PS complex, showing a concentration-dependent relationship between PS concentration and SMG remaining in solid-state form (%) in PBS buffer (Figure 2b). In the absence of Zn, the proportion of solid-state SMG in PBS (%) increased from 17% at 0.2 mg/mL PS to 88% at 1.2 mg/mL PS, indicating enhanced complex formation and lowered solubility at higher PS concentrations. The presence of Zn (0.5 mg/mL) further augmented the proportion of solid-state SMG (%), with values reaching 18% at 0.2 mg/mL PS and complete precipitation at 1.2 mg/mL PS. This behavior aligns with established principles of protamine–peptide interactions, where multiple arginine residues in protamine form ionic complexes with anionic peptides [40]. The concentration-dependent increase in precipitation reflects the formation of progressively larger and less soluble complexes as more protamine molecules associate with SMG. The enhanced precipitation in the presence of Zn is attributed to zinc’s ability to coordinate with both peptide components, similar to the well-characterized zinc–insulin–protamine systems used in sustained-release formulations [41].

### 3.2. Effect of Suspending Agent on Size, Re-Dispersibility, and Solubility of SMG-PS-Zn Nanocomplexes

**Figure 3:**
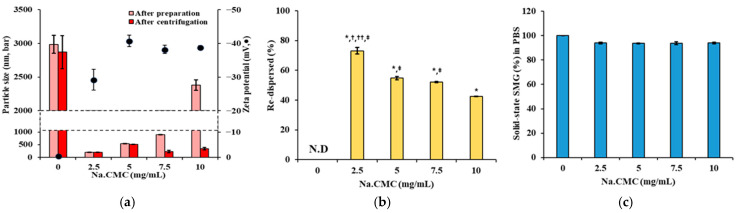

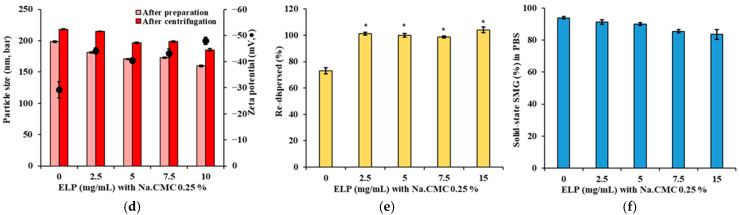
Effects of suspending agents on particle size, surface charge, redispersibility, and the proportion of SMG in solid-sate (%) of SMG–PS–Zn complexes in PBS at 25 °C. (**a**) Particle size and zeta potential of complexes formulated with varying Na.CMC concentrations (0–10 mg/mL). (**b**) Redispersibility (%) of the complexes after centrifugation at 13,000 rpm. (**c**) The proportion of SMG in solid-sate (%) in PBS following dilution of complexes prepared with different Na.CMC concentrations. (**d**) Particle size and zeta potential of complexes prepared with varying concentrations of Kolliphor ELP (0–10 mg/mL) and 2.5 mg/mL Na.CMC. (**e**) Redispersibility (%) after centrifugation, and (**f**) The proportion of SMG in solid-sate (%) in PBS of complexes prepared with Kolliphor ELP/Na.CMC combinations. Notes: The concentration of SMG, PS, and Zn was set to 5.36, 1.2, 0.5, mg/mL, respectively. Data represents mean ± SD (n = 3). N.D means ‘not detected’. In panel (**b**), significant differences compared with Na.CMC 0 mg/mL (* *p* < 0.05), 5 mg/mL (^†^ *p* < 0.05), 7.5 mg/mL (^††^ *p* < 0.05), and 10 mg/mL (^‡^ *p* < 0.05), respectively. In panel (**e**), significant difference compared with ELP 0 mg/mL (* *p* < 0.05). In panels (**c**,**f**), no significant differences (*p* > 0.05) between formulations.

Na.CMC, alone and in combination with Kolliphor ELP, was evaluated as a suspending agent to control the particle size and enhance the dispersion stability of the SMG–PS–Zn complex. The concentrations of SMG, PS, and Zn were fixed at 5.36, 1.2, and 0.5 mg/mL, respectively. As shown in Figure 3a, a biphasic relationship was observed between Na.CMC concentration and particle size. In the absence of Na.CMC (0 mg/mL), the particle size was approximately 3000 nm, indicating significant aggregation and poor colloidal stability. Upon addition of 2.5 mg/mL Na.CMC, the particle size dramatically decreased to ~200 nm, representing a 15-fold reduction. Further increasing Na.CMC to 5.0 mg/mL maintained particle sizes between 150–200 nm. At low concentrations (2.5–7.5 mg/mL), Na.CMC provided effective electrostatic stabilization by adsorbing onto particle surfaces and generating repulsive forces that prevented aggregation [42]. The sharp reduction in size from 3000 nm to 200 nm suggests successful dispersion of large aggregates via electrostatic repulsion and steric hindrance of the cellulose derivative. However, at 10 mg/mL Na.CMC, particle size increased to ~400 nm, probably due to bridging flocculation. This phenomenon occurs when polymer chains exceed optimal surface coverage and simultaneously adsorb onto multiple particles, forming interparticle bridges [43]. Centrifugation data further supported this, showing enhanced aggregation at suboptimal Na.CMC levels (0 or 10 mg/mL). Zeta potential values ranged from −30 to −50 mV across all Na.CMC concentrations, indicating moderate to strong electrostatic repulsion. Redispersibility studies (Figure 3b) revealed poor colloidal stability of nanocomplexes with Na.CMC alone. Resuspended drug content decreased from 75% at 2 mg/mL to 42% at 5 mg/mL, suggesting irreversible aggregation during centrifugation. This likely results from strong particle–particle interactions that Na.CMC alone cannot overcome. Solubility studies in PBS (Figure 3c) showed a consistently high proportion of SMG as solid-state (>90%) across all Na.CMC concentrations, confirming that the complex remained poorly water-soluble regardless of Na.CMC content, even after addition into PBS.

To address the physical stability issue of the nanocomplex, Kolliphor ELP was introduced as a co-suspending agent at 2.5–15 mg/mL alongside 0.25% Na.CMC. This combination (Figure 3d) provided superior particle size control, with minimal changes before and after centrifugation, maintaining sizes around 200 nm. Zeta potential remained stable (−45 to −50 mV), indicating preserved electrostatic stabilization. Most notably, redispersibility improved dramatically (Figure 3e), with >95% resuspension across all Kolliphor ELP concentrations. As a nonionic surfactant, Kolliphor ELP offers steric stabilization by forming a protective barrier around particles, complementing the electrostatic effects of Na.CMC [44,45]. This dual mechanism prevents irreversible aggregation under mechanical stress and maintains high proportion of SMG as solid-state in PBS (Figure 3f), confirming that suspending agents do not interfere with complex formation while enhancing physical stability for pharmaceutical processing.

### 3.3. Optimization of SMG-PS-Zn Complexes

**Figure 4:**
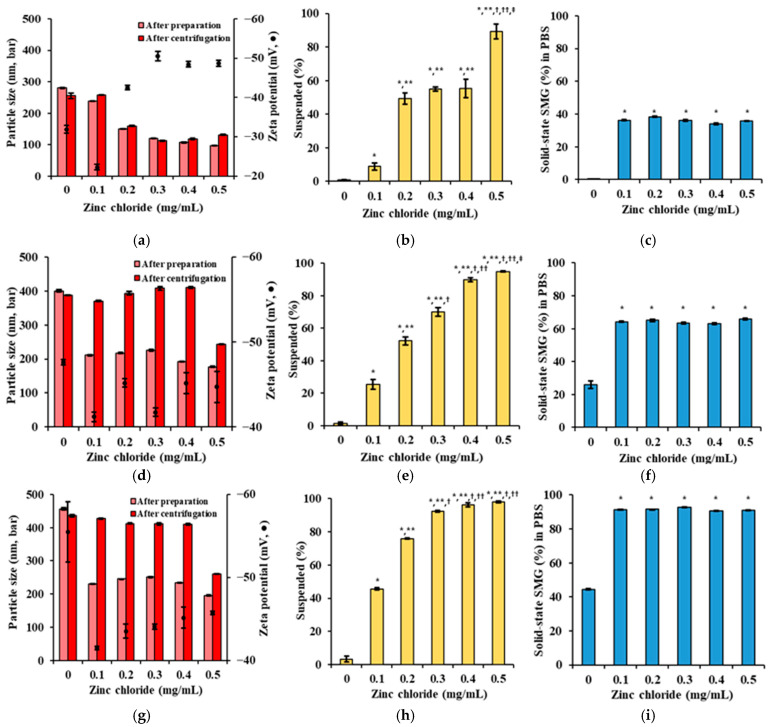
Effects of PS and Zn concentrations on particle size, surface charge, suspended drug content (%), and drug precipitation (%) of SMG–PS–Zn nanocomplexes in PBS at 25 °C. (**a**) Particle size and zeta potential of complexes formulated with 0.4 mg/mL PS and varying Zn concentrations (0–0.5 mg/mL). (**b**) Suspended drug content (%) of SMG–PS–Zn complexes in the vehicle at corresponding PS and Zn levels. (**c**) The proportion of SMG in solid-state (%) in PBS following dilution of the above complexes. (**d**–**f**) Same analyses as panels (**a**–**c**) for complexes prepared with 0.8 mg/mL PS and different Zn concentrations (0–0.5 mg/mL). (**g**–**i**) Same analyses for complexes prepared with 1.2 mg/mL PS and varying Zn concentrations (0–0.5 mg/mL). Notes: Data represents mean ± SD (n = 3). In panels (**b**,**e**,**h**), significant differences compared with ZnCl_2_ 0 mg/mL (* *p* < 0.05), 0.1 mg/mL (** *p* < 0.05), 0.2 mg/mL (^†^ *p* < 0.05), 0.3 mg/mL (^††^ *p* < 0.05), and 0.4 mg/mL (^‡^ *p* < 0.05), respectively. In panels (**c**,**f**,**i**), significant difference compared with ZnCl_2_ 0 mg/mL (* *p* < 0.05).

The ratio of SMG, PS, and Zn was further fine-tuned with respect to particle size, solid-state SMG (%) in the formulation, and upon dilution in PBS [46]. The concentrations of SMG, Kolliphor ELP, and Na.CMC were fixed at 5 mg/mL, 2 mg/mL, and 2.5 mg/mL, respectively, while PS (0.4, 0.8, 1.2 mg/mL) and Zn (0–0.5 mg/mL) concentrations were varied. The results demonstrated that increasing PS and Zn concentrations significantly influenced the physical properties and suspension stability of the SMG–PS–Zn nanocomplex (Figure 4). At the lowest PS concentration (0.4 mg/mL), particle sizes were initially small but increased after centrifugation, indicating poor mechanical stability and a tendency toward aggregation under stress. As Zn concentration increased, the proportion of dissolved SMG decreased while the suspended fraction rose; however, a substantial portion remained dissolved, PBS precipitation remained between 40–50% (Figure 4a–c).

At 0.8 mg/mL PS, particle sizes after both preparation and centrifugation were more consistent (200–400 nm), reflecting improved colloidal stability (Figure 4d–f). The suspended fraction increased markedly with higher Zn, while the dissolved fraction decreased, suggesting greater incorporation of SMG into low-solubility complexes. Correspondingly, PBS precipitation increased to 60–70%, indicating enhanced formation of insoluble nanocomplexes. This behavior aligns with previous findings that higher protamine content promotes more complete ionic crosslinking with peptides, thereby stabilizing the nanocomplex and reducing aqueous solubility [47,48].

At the highest PS concentration tested (1.2 mg/mL), the nanocomplexes exhibited robust physical stability, with particle sizes remaining steady before and after centrifugation (300–400 nm) (Figure 4g–i). The suspended fraction approached 100% as Zn increased, while the dissolved fraction was minimized. The proportion of SMG in solid-state (%) reached up to 90%, indicating near-complete conversion of SMG into a stable, insoluble nanocomplex (Figure 4c,f,i). This trend supports the strategy of using excess protamine to ensure full complexation and optimal physical stability, consistent with peptide–polyelectrolyte systems designed for sustained release [49].

Across all PS concentrations, increasing Zn consistently enhanced the suspended fraction in the vehicle and even after addition into PBS, underscoring Zn’s stabilizing role in nanocomplex formation [50]. The zeta potential remained strongly negative (−30 to −50 mV), indicative of effective electrostatic stabilization, which is essential for maintaining nanoscale dispersion and preventing aggregation. Collectively, these findings suggest that higher PS and Zn content synergistically promote the formation of stable, insoluble SMG–PS–Zn nanocomplexes—an advantageous strategy for developing SC sustained-release peptide formulations by minimizing initial burst release and enhancing solid-state depot formation at the injection site.

### 3.4. Interaction Between SMG, PS and Zn in Complexes

**Figure 5:**
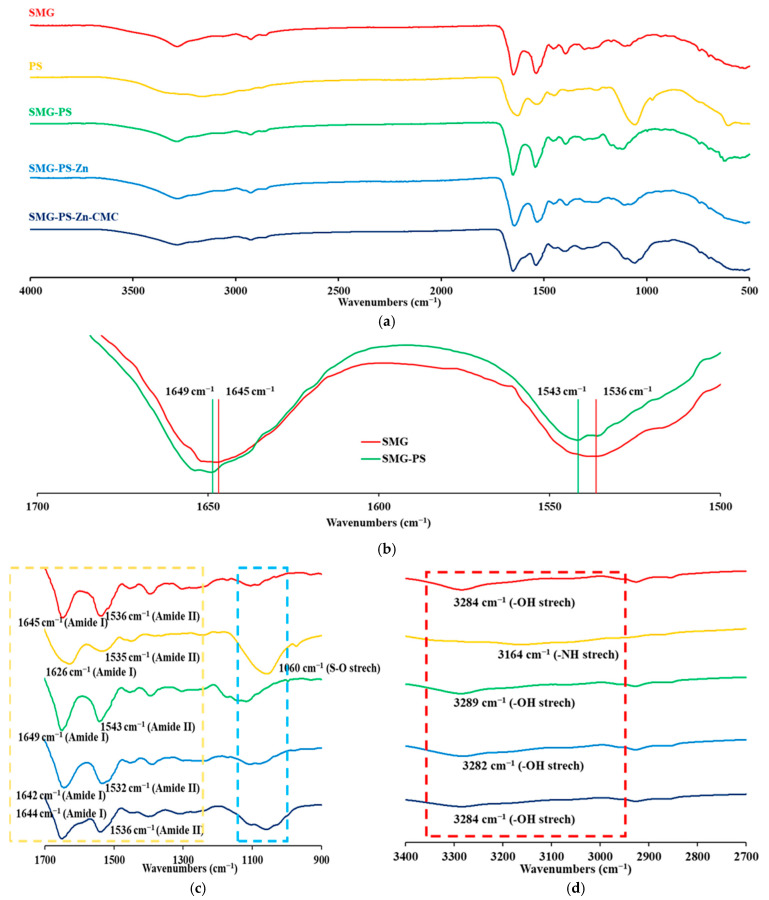
FT-IR spectra of SMG, PS, and their electrostatic complexes prepared at various weight ratios: SMG–PS (5.36:1.2), SMG–PS–Zn (5.36:1.2:0.5), and SMG–PS–Zn–CMC (5.36:1.2:0.5:2.5). (**a**) Full spectral range from 500 to 4000 cm^−1^. (**b**) Expanded region highlighting the shifts in the Amide I and II bands. (**c**) Fingerprint region showing amide I and amide II bands, along with additional characteristic peaks indicating newly formed bonding interactions. (**d**) Expanded region highlighting hydroxyl (O–H) and protonated amine (NH_3_^+^) stretching vibrations. Note: Dashed boxes mark key absorption bands of interest. Remarkable wavenumbers were denoted in the FT-IR spectrum.

The interaction between SMG and PS, along with Zn and Na.CMC was investigated using FT-IR spectroscopy. Both SMG and PS exhibited characteristic amide I (C=O stretching, ~1650–1652 cm^−1^) and amide II (N–H bending, ~1540–1550 cm^−1^) bands (Figure 5a). Upon complex formation, the amide I (1645 → 1649 cm^−1^) and amide II (1536 → 1543 cm^−1^) bands shifted, indicating changes in hydrogen bonding environments and potential conformational rearrangements (Figure 5b) [51,52,53]. These few-cm^−1^ shifts of amide bands are well documented as qualitative indicators of altered hydrogen-bonding and electrostatic microenvironments in proteins/peptides [54,55,56]. The narrowing of these bands was further observed in the enlarged FT-IR spectra, reflecting conformational heterogeneity and local dynamics, and greater microenvironmental homogeneity/constraint upon complexation [57,58]. A distinct PS band near ~1049–1050 cm^−1^, which originates from sulfate/bisulfate vibrations [59], was not observed in SMG–PS complex as sulfate molecules were dissociated and released into the bulk solution, during the interaction between protamine and SMG (Figure 5c). Furthermore, the O–H stretching region (3100–3300 cm^−1^) in SMG exhibited shifts upon complexation with PS, indicating the involvement of hydroxyl groups in hydrogen bonding or ionic interactions (Figure 5d). In contrast, the N–H stretching band of PS in the same region disappeared in the SMG–PS complex, suggesting strong ionic interactions involving the protonated amine groups of PS. These observations provide additional evidence for robust electrostatic interactions between the two components.

The addition of Zn further altered the amide I and II regions (Figure 5c), with additional shifts and intensity changes reflecting coordination between Zn^2+^ and peptide functional groups. Zinc is known to coordinate with carboxylate (–COO^−^) and imidazole (from histidine) side chains, leading to a shift of the amide I band to lower wavenumbers and possible broadening of the band—indicative of altered hydrogen bonding and metal–ligand interactions [35]. Notably, in the presence of Zn, strong low-wavenumber shifts, and broadening were observed not only in the amide bands but also across O–H containing regions, suggesting the formation of a distinct SMG–PS–Zn complex with enhanced coordination bonding compared to the binary SMG–PS system (Figure 5c,d).

Interestingly, in the nanocomplex containing Na.CMC, the previously observed shifts were partially reversed, implying that CMC influenced the binding and formation of SMG–PS–Zn complex (Figure 5c,d). This interference appears to weaken the original SMG–PS–Zn interactions. A decrease in intensity was observed in the amide II region, which may reflect additional interactions between CMC and the amide backbone of SMG, indicating the formation of new, possibly stabilizing interactions within the nanocomplex system.

### 3.5. Morphological and Physical Characteristics of SMG-PS-Zn Nanocomplexes

**Figure 6:**
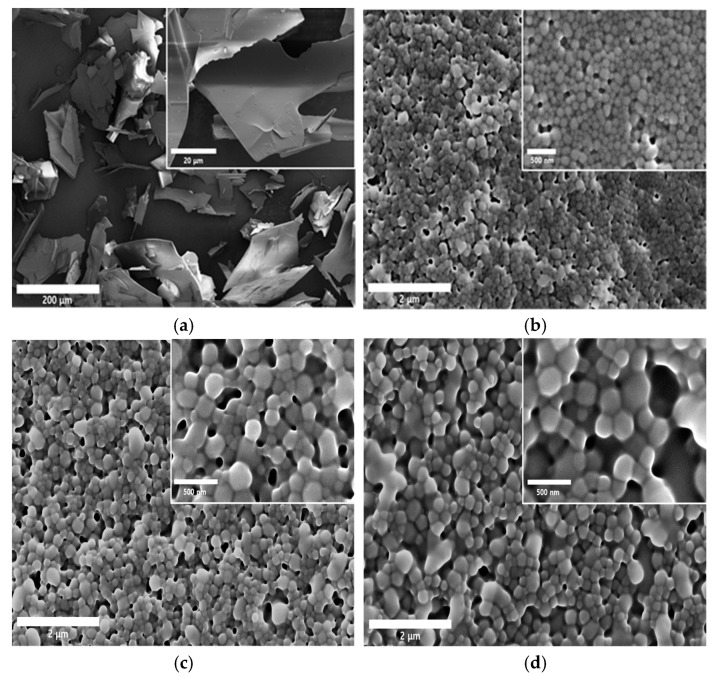

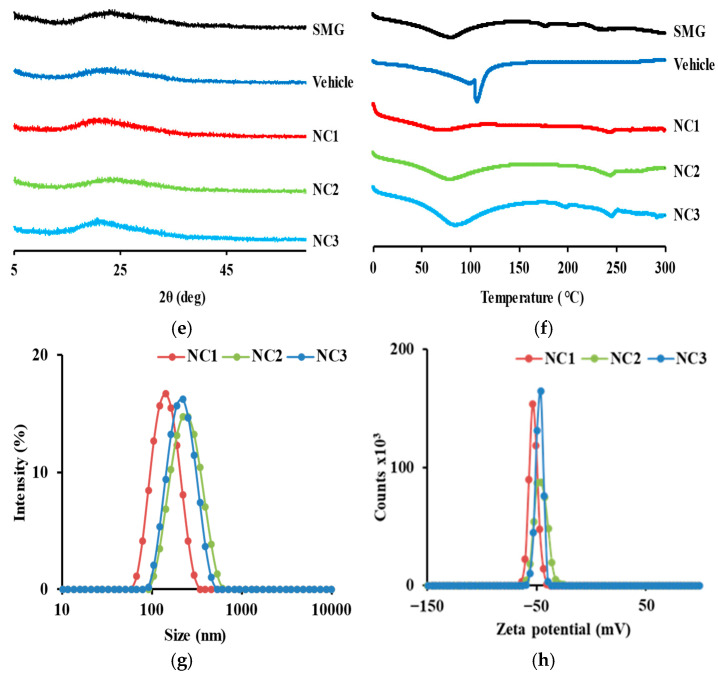
Representative SEM images of SMG raw material and SMG–PS–Zn nanocomplexes. (**a**) SMG raw material; (**b**) SMG–PS–Zn nanocomplex prepared at a 5.36:0.4:0.5 ratio (NC1); (**c**) nanocomplex at a 5.36:0.8:0.5 ratio (NC2); and (**d**) nanocomplex at a 5.36:1.2:0.5 ratio (NC3). Physical characteristics of SMG-PS-Zn nanocomplexes. (**e**) XRD patterns and (**f**) DSC curves of SMG raw material, blank vehicle, and nanocomplexes respectively. (**g**) Particle size distributions and (**h**) zeta potential profiles of the nanocomplexes (NC1, NC2, and NC3).

The SMG API alone exhibited a typical film-like morphology commonly observed in dried peptide materials (Figure 6a). Upon the addition of PS, electrostatic interactions between the positively charged PS and the negatively charged SMG led to the formation of nanoparticles. Regardless of PS concentration, all nanocomplexes (NC1, NC2, and NC3) exhibited similar spherical nanoparticle morphology (Figure 6b–d). As the concentration of PS increased, particle size gradually rose from approximately 150 nm to the 250 nm range.

XRD analysis (Figure 6e) confirmed that raw SMG exhibits an amorphous structure, characterized by broad halos and the absence of sharp crystalline peaks [60,61]. All nanocomplex formulations (NC1–NC3) retained this amorphous nature, suggesting that complexation does not induce crystallization but instead promotes disordered molecular arrangements. DSC analysis (Figure 6f) supported these findings, displaying broad thermal transitions without distinct melting points, further confirming the amorphous character.

In DLS-based particle size analysis (Figure 6g), a shift toward larger particle dimensions was observed with increasing PS concentration, consistent with SEM results. Zeta potential measurements (Figure 6h) showed highly negative surface charges across all formulations (−40 to −60 mV), indicating strong electrostatic stabilization that prevents aggregation during storage [62,63]. Notably, the negative surface charge was maintained despite the addition of cationic protamine, suggesting that hydrophobic domains formed via ionic interactions between SMG and PS are sequestered within the particle core, while negatively charged groups—such as carboxylates from SMG and Na.CMC— are exposed on the surface. This structural arrangement promotes colloidal stability by enabling favorable interactions with the aqueous environment.

### 3.6. In Vitro Dissolution Profile of SMG–PS–Zn Nanocomplexes

The in vitro dissolution profile of SMG from the nanocomplexes was evaluated using the dialysis bag method, which is widely recognized as a suitable approach for colloidal drug carriers and peptide-based formulations [64]. This method minimizes interference from suspended particles during sampling and enables accurate assessment of free drug release [65]. Dialysis bags with a MWCO of 1000 kDa were used to retain nanocomplex particles while allowing diffusion of free SMG. In our preliminary release study using native SMG, no significant differences in the release profiles were observed between the two membrane pore sizes, 100 kDa and 1000 kDa. The bags were immersed in 200 mL of PBS (pH 7.4) as the release medium, which provided sufficient solubility (26.8 mg/mL) to maintain sink conditions.

**Figure 7:**
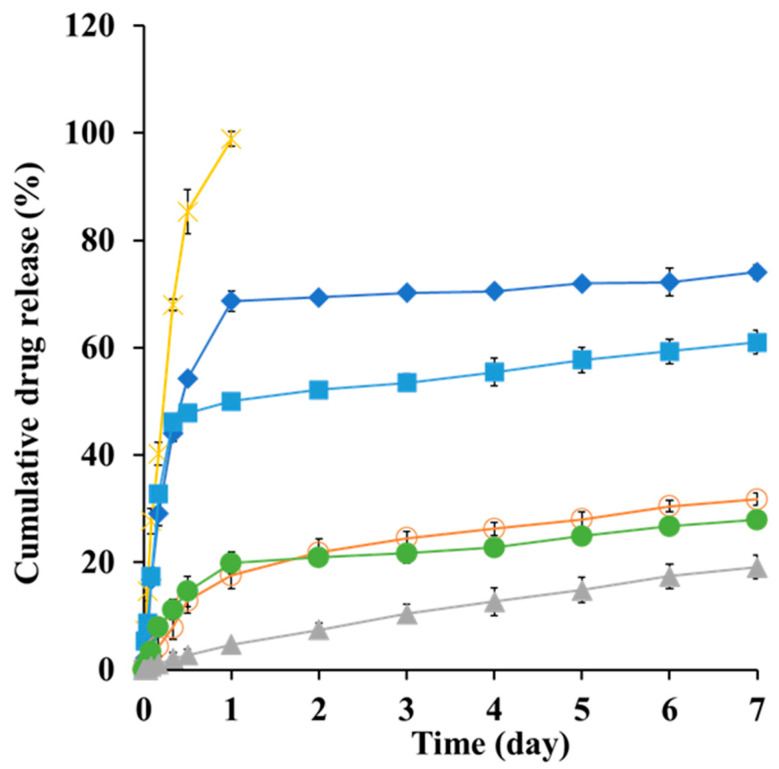
In vitro dissolution profiles of SMG raw material (X) and SMG–PS–Zn nanocomplexes with varying SMG:PS:Zn ratios 5.36:0.4:0.5 (NC1, ♦), 5.36:0.8:0 (■), 5.36:0.8:0.3 (●), 5.36:0.8:0.5 (NC2, ○), and 5.36:1.2:0.5 (NC3, ▲), respectively in PBS (pH 7.4, 37 °C) using a dialysis bag model. Note: Data represent mean ± SD (n = 3).

Under these conditions, a clear concentration-dependent relationship was observed between PS and Zn content and SMG release kinetics. Native SMG exhibited rapid dissolution, achieving 98% release within 24 h (Figure 7), consistent with the immediate-release behavior of unformulated hydrophilic peptides [66]. In contrast, nanocomplexes containing PS showed significantly delayed release profiles. For instance, NC1 (0.4 mg/mL PS and 0.5 mg/mL Zn) released 73% of SMG over 7 days, whereas NC3 (1.2 mg/mL PS and 0.5 mg/mL Zn) released only 19% over the same period. This progressive reduction in release rate with increasing PS concentration is attributed to enhanced ion pairing between cationic protamine and anionic SMG, resulting in the formation of more condensed, stable complexes with reduced aqueous solubility [67].

Interestingly, the addition of Zn further retarded drug release. For example, in formulations containing 0.8 mg/mL PS, the Zn-free complex released 62% of SMG, whereas formulations with 0.3 and 0.5 mg/mL Zn released only 31% and 27%, respectively (Figure 7). This enhanced sustained release effect is consistent with previous findings that Zn coordinates with peptide residues, thereby stabilizing protein–protamine complexes through additional cross-linking mechanisms [35,67]. The formation of larger, more hydrophobic nanostructures at higher PS and Zn concentrations aligns with physical characteristics and supports the hypothesis that increased hydrophobicity correlates with prolonged release duration.

This study introduced a novel electrostatic nanocomplex system of SMG designed for prolonged SC delivery, through comprehensive compositional exploration, physicochemical characterization, interaction analysis, and in vitro dissolution test. In contrast to a conventional biodegradable polymer-based sustained-release system, this approach represents the first attempt to regulate drug release primarily through reduced solubility induced by complex formation, rather than relying on physical barriers. This strategy presented the potential to overcome the limitations of existing biodegradable polymer-based formulations, such as low encapsulation efficiency, incomplete drug release, and low drug stability. According to a previous report [68], poly(lactic-co-glycolic acid) microspheres prepared via the double emulsion method achieved over 90% encapsulation efficiency, by employing a polymer-to-drug ratio of 30:1 *w*/*w*. However, only 27.2% of the drug was cumulatively released by final day 44, with nearly 50% degraded or lost during dissolution test, highlighting the need for alternative controlled-release systems. However, our dissolution test has limitations in simulating subcutaneous environments, particularly with respect to enzymatic hydrolysis and protein adsorption. The GLP-1 agonist has been reported to undergo metabolism across various tissues via proteolytic cleavage of the peptide backbone, followed by sequential β-oxidation of the fatty diacid side chain [69]. Moreover, the strong affinity of SMG for proteins, including albumin, reduces its degradation in the subcutaneous milieu and contributes to its extended biological half-life [70]. To address these limitations, further studies are planned to investigate enzymatic degradation, preclinical pharmacokinetics and pharmacodynamics, as well as analyses of the binding sites and ratios between SMG and cationic molecules (PS or Zn), viscoelasticity, and physicochemical stability of the nanocomplexes under storage conditions.

## 4. Conclusions

The electrostatic nanocomplex system of SMG was successfully designed employing a cationic polypeptide (PS) and a metallic coordination agent (Zn) for SC prolonged delivery. Increasing PS and Zn enhanced the lipophilicity of the SMG–PS–Zn complexes as evidenced by a shift in logP. The use of dual suspending agents, Kolliphor ELP and Na.CMC provided stable solid-state dispersion of the nanocomplex through complementary steric and electrostatic mechanisms, ensuring excellent re-dispersibility. In an in vitro dissolution under sink conditions, the optimized nanocomplex (SMG:PS:Zn = 5.36:1.2:0.5 *w*/*w*/*w*) exhibited marked sustained release with only 19% drug release over the 7 days, whereas unformulated SMG was dissolved completely within 24 h. From these findings, this electrostatic nanocomplex system is expected to be a promising tool for long-acting injectable SMG, potentially improving patient compliance and therapeutic efficacy in diabetes and obesity management, after further investigations.

## Data Availability

The data that support the findings of this study are available from the corresponding author upon reasonable request.
